# Supplementary figures and images for: Ante- and post-mortem cellular injury dynamics in hybrid poplar foliage as a function of phytotoxic O3 dose
Source: PLoS One. 2023 Mar 1;18(3):e0282006. doi: 10.1371/journal.pone.0282006 (PMC9977006; doi:10.1371/journal.pone.0282006)

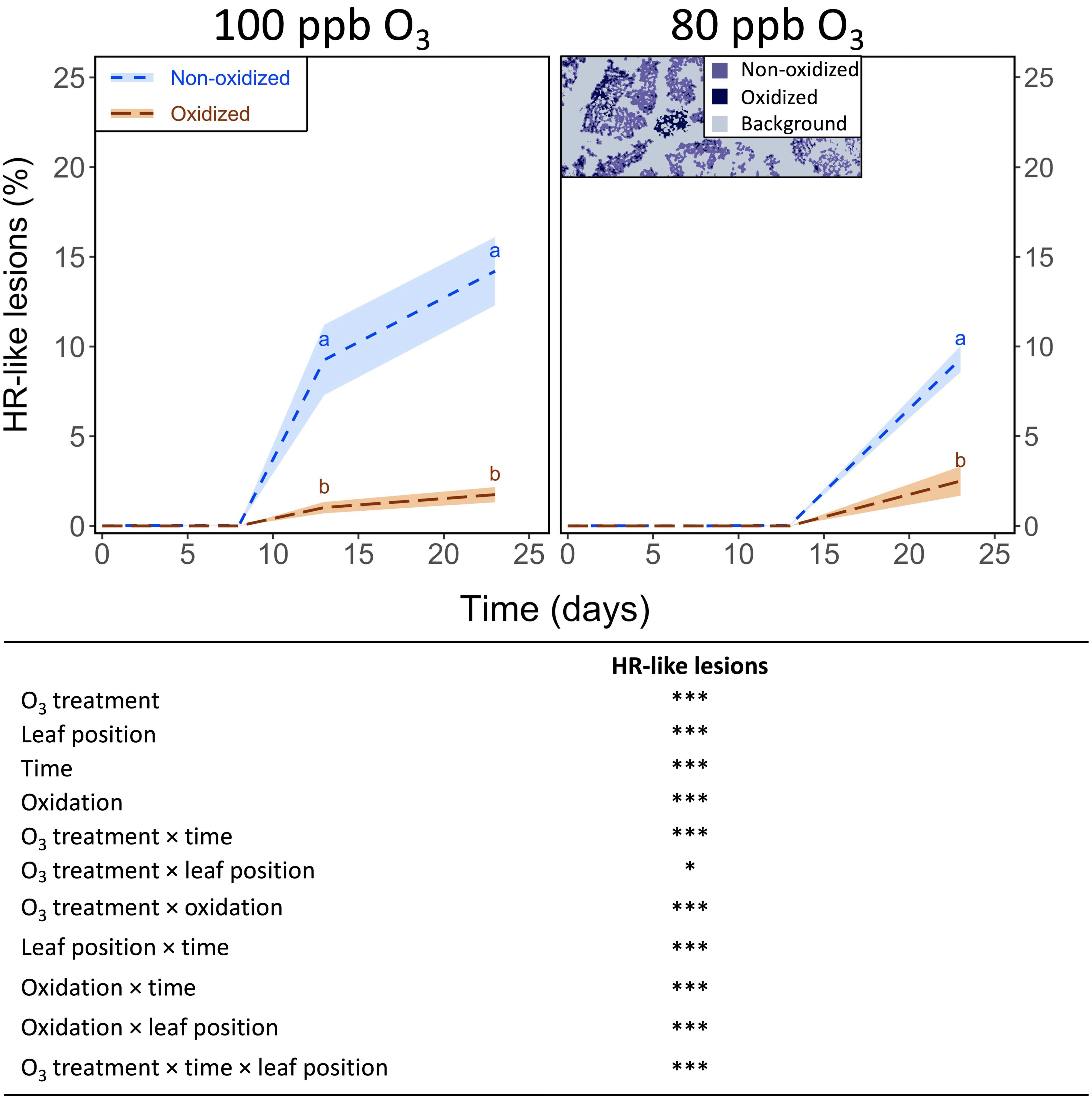

Supplement: S1 Fig — {model: lmer[log(variable+1) ~ leaf position * oxidation * time * O3 treatment + (1|pot)]; *** P ≤ 0.001; * P ≤ 0.05}.}. The inset image is a synthetic image of the particle distribution and morphology in each lesion color class (non-oxidized/oxidized) during image analyses of HR-like reactions. Values represent percentage area means ± SE of leaf discs showing non-oxidized or oxidized HR-like lesions (n = 4). Different letters indicate significant differences between treatments at a given assessment date (Tukey’s honestly significant difference post-hoc test, P ≤ 0.05). (TIFF) [file pone.0282006.s001.tiff]

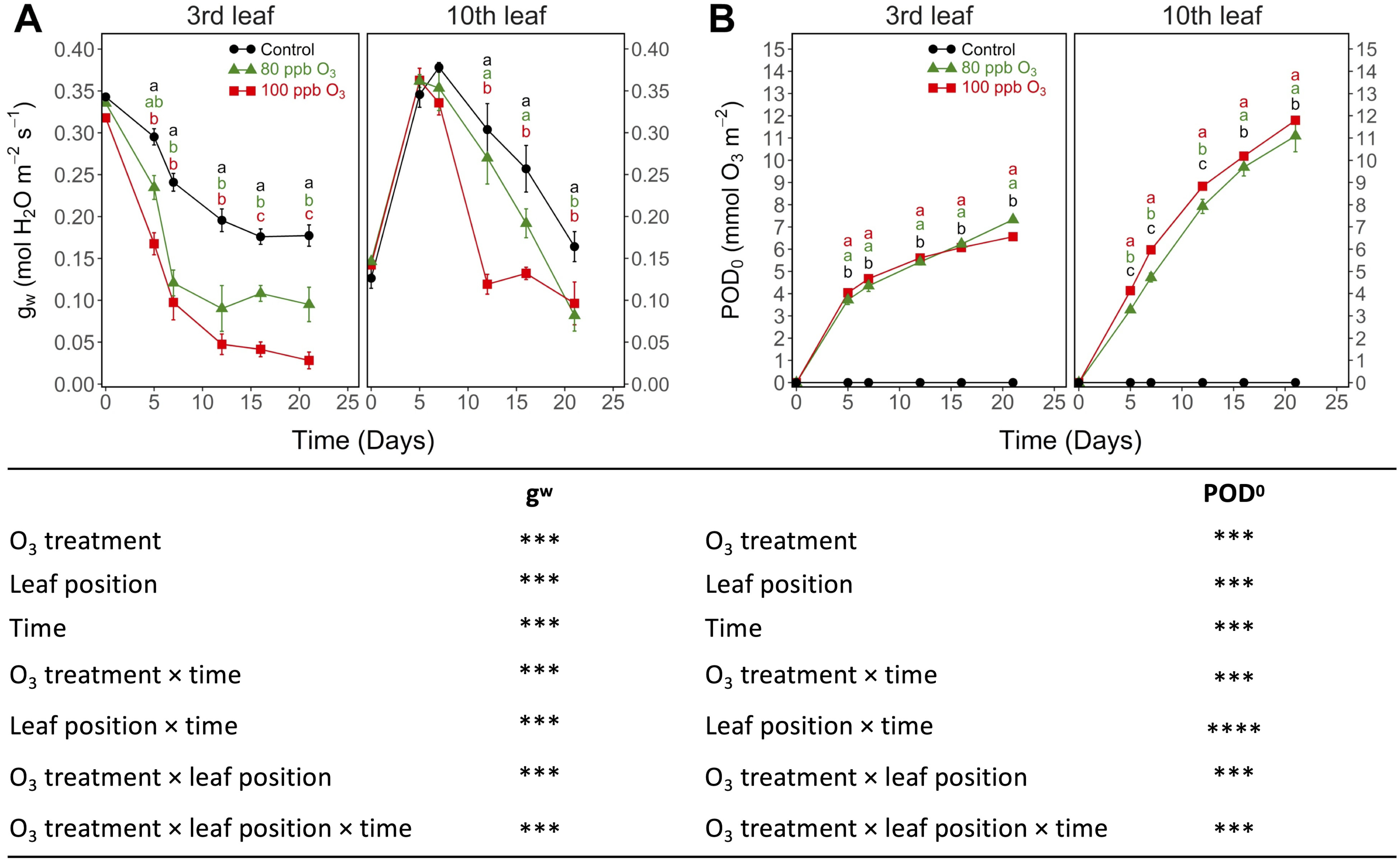

Supplement: S2 Fig — {model: lmer[sqrt(variable)] ~ O3 treatment * leaf position * time + (1 | tree/chamber); ***P ≤ 0.001}. Values represent means ± SE (n = 4). Different letters indicate significant differences between treatments for a given assessment date (Tukey’s honestly significant difference post-hoc test, P ≤ 0.05). (TIFF) [file pone.0282006.s002.tiff]

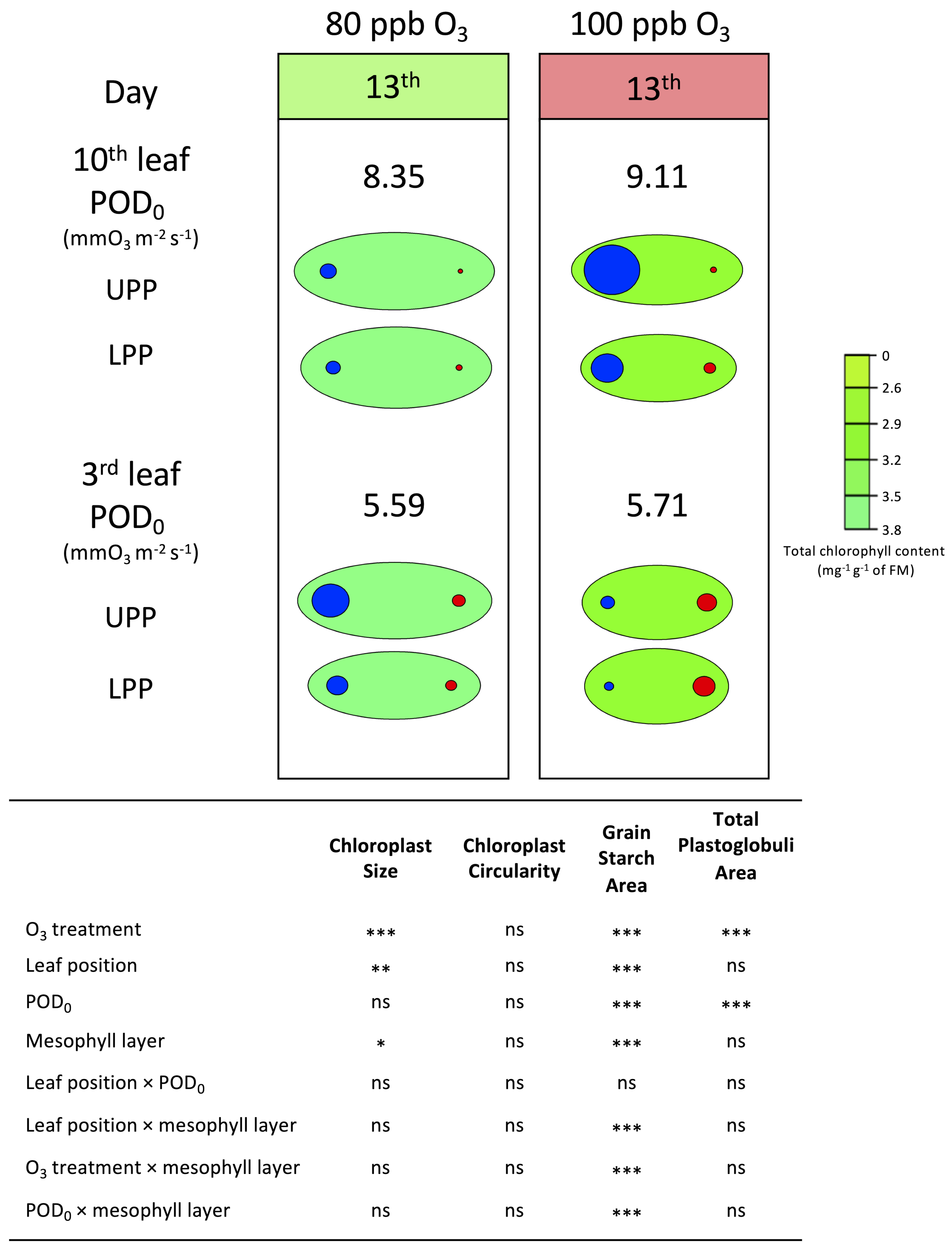

Supplement: S3 Fig — Model: lmer(variable ~ O3 treatment * leaf position * POD0 + (1 | cell/tree)); *** P ≤ 0.001, * P ≤ 0.05, ns not significantly different). Green ellipse: chloroplast, blue circle: total starch grain area, red circle: total plastoglobuli area. Green shading represents the total chlorophyll content (mg g-1 of FM). (TIFF) [file pone.0282006.s003.tiff]
