# Supplementary material for: Ante- and post-mortem cellular injury dynamics in hybrid poplar foliage as a function of phytotoxic O3 dose
Source: PLoS One. 2023 Mar 1;18(3):e0282006. doi: 10.1371/journal.pone.0282006 (PMC9977006; doi:10.1371/journal.pone.0282006)
Supplement: S1 Table — Values represent mean ± SE, n = 4. Different letters indicate significant differences between treatments for a given assessment date (Tukey’s honestly significant difference post-hoc test, P ≤ 0.05). (model implemented in R: lmer(variable ~ O3 treatment * leaf position * time + mesophyll layer +(1 | cell/tree)). (DOCX) [file pone.0282006.s004.docx]

|  |  | Charcoal-filtered air | | | | | | | | 80 ppb O_3_ | | | | | | | | 100 ppb O_3_ | | | | | | | |
| --- | --- | --- | --- | --- | --- | --- | --- | --- | --- | --- | --- | --- | --- | --- | --- | --- | --- | --- | --- | --- | --- | --- | --- | --- | --- |
|  |  | 3^rd^ leaf position | | | | 10^th^ leaf position | | | | 3^rd^ leaf position | | | | 10^th^ leaf position | | | | 3^rd^ leaf position | | | | 10^th^ leaf position | | | |
| Chloroplast trait (µm²) | Exposure  (days) | Upper  Palisade  Parenchyma | | Lower  Palisade  Parenchyma | | Upper  Palisade  Parenchyma | | Lower  Palisade  Parenchyma | | Upper  Palisade  Parenchyma | | Lower  Palisade  Parenchyma | | Upper  Palisade  Parenchyma | | Lower  Palisade  Parenchyma | | Upper  Palisade  Parenchyma | | Lower  Palisade  Parenchyma | | Upper  Palisade  Parenchyma | | Lower  Palisade  Parenchyma | |
| Chloroplast area | 8 | 12.71 ±0.88 | a | 13.54 ±0.81 | a | 14.67 ±0.86 | a | 12.97 ±0.61 | a | NA |  | NA |  | NA |  | NA |  | 14.07  ±1.11 | a | 11.91 ±0.55 | a | 13.86 ±0.68 | a | 13.74 ±0.53 | a |
|  | 13 | NA |  | NA |  | NA |  | NA |  | 12.92 ±0.70 | bcd | 10.23 ±0.61 | abc | 13.55 ±0.84 | d | 13.25 ±0.74 | cd | 10.18  ±0.53 | abcd | 9.93 ±0.73 | ab | 10.70 ±0.63 | abcd | 9.3 ±0.71 | a |
|  | 23 | 8.84 ±0.51 | a | 8.97 ±0.39 | a | 10.78 ±0.72 | a | 8.75 ±0.47 | a | NA |  | NA |  | NA |  | NA |  | 8.07  ±0.63 | a | 8.11 ±0.43 | a | 9.04 ±0.61 | a | 9.35 ±0.60 | a |
| Chloroplast circularity | 8 | 0.55 ±0.02 | ab | 0.6 ±0.03 | abc | 0.62 ±0.01 | abc | 0.63 ±0.02 | abc | NA |  | NA |  | NA |  | NA |  | 0.54  ±0.04 | a | 0.63 ±0.0 | bc | 0.69 ±0.02 | c | 0.71 ±0.02 | c |
|  | 13 | NA |  | NA |  | NA |  | NA |  | 0.63 ±0.02 | ab | 0.63 ±0.02 | ab | 0.63 ±0.02 | a | 0.61 ±0.02 | ab | 0.65  ±0.03 | b | 0.64 ±0.02 | b | 0.73 ±0.02 | ab | 0.72 ±0.03 | ab |
|  | 23 | 0.53 ±0.02 | a | 0.57 ±0.02 | ab | 0.57 ±0.01 | ab | 0.54 ±0.02 | ab | NA |  | NA |  | NA |  | NA |  | 0.68  ±0.02 | bc | 0.73 ±0.02 | d | 0.77 ±0.02 | cd | 0.79 ±0.02 | cd |
| Total starch grain area^1^ | 8 | 0.16 ±0.06 | a | 0.19 ±0.07 | a | 3.84 ±0.39 | bd | 2.21 ±0.29 | ac | NA |  | NA |  | NA |  | NA |  | 1.7  ±0.47 | abcd | 1.32 ±0.3 | ab | 3.39 ±0.31 | cd | 2.36 ±0.33 | ab |
|  | 13 | NA |  | NA |  | NA |  | NA |  | 0.65 ±0.15 | bc | 0.18 ±0.06 | a | 0.12 ±0.04 | a | 0.09 ±0.04 | a | 0.06  ±0.04 | a | 0.03 ±0.03 | a | 1.16 ±0.17 | c | 0.35 ±0.09 | ab |
|  | 23 | - | - | - | - | - | - | - | - | - | - | - | - | - | - | - | - | - | - | - | - | - | - | - | - |
| Total plastoglobuli area | 8 | 0.05 ±0.007 | bc | 0.05 ±0.01 | c | 0.01 ±0.004 | ab | 0.03 ±0.005 | abc | NA |  | NA |  | NA |  | NA |  | 0.02  ±0.01 | ab | 0.01 ±0.004 | ab | 0.00 ±0.0007 | a | 0.00 ±0.002 | ab |
|  | 13 | NA |  | NA |  | NA |  | NA |  | 0.07 ±0.01 | c | 0.04 ±0.006 | c | 0.008 ±0.003 | a | 0.02 ±0.005 | ab | 0.12  ±0.015 | d | 0.15 ±0.016 | d | 0.01 ±0.004 | a | 0.04 ±0.005 | bc |
|  | 23 | 0.05 ±0.008 | a | 0.05 ±0.007 | a | 0.02 ±0.004 | a | 0.02 ±0.005 | a | NA |  | NA |  | NA |  | NA |  | 0.22  ±0.04 | b | 0.23 ±0.02 | b | 0.23 ±0.03 | b | 0.16 ±0.02 | b |

**Table S1.** Evolution of chloroplast size and shape in the leaf mesophyll of hybrid poplar trees (*Populus tremula* x *alba*) in response to O_3_ treatment, leaf position, mesophyll layer and assessment time (model implemented in R: lmer(variable ~ O_3_ treatment * leaf position * time + mesophyll layer +(1 | cell/tree)). Values represent mean ± SE, n = 4. Different letters indicate significant differences between treatments for a given assessment date (Tukey’s honestly significant difference post-hoc test, *P* ≤ 0.05).

^1^Starch grains missing in samples treated for 23 days
