# Supplementary material for: Ante- and post-mortem cellular injury dynamics in hybrid poplar foliage as a function of phytotoxic O3 dose
Source: PLoS One. 2023 Mar 1;18(3):e0282006. doi: 10.1371/journal.pone.0282006 (PMC9977006; doi:10.1371/journal.pone.0282006)
Supplement: Attachment — Submitted filename: Response to Reviewer.docx [file pone.0282006.s005.docx]

From Benjamin TURC, (corresponding author)

UMR 1434 Silva Université de Lorraine, AgroParisTech, INRAE

Faculté des Sciences et Technologies

BP70239

F-54506 Vandoeuvre-lès-Nancy Cedex, France

Phone: +33 3 72 74 51 87

Email : benjamin.turc@univ-lorraine.fr

Lincoln, 5^th^ February 2023

Dear reviewer and editor,

Regarding non-destructive reflectance-based chlorophyll estimation (line 389): I agree and added your suggestion to the manuscript. Reflectance-based estimation is a very good way to estimate chlorophyll content, and has this advantage to be non-destructive, allowing multiple measurements on the same leaf along stress. We used this method in the first experiment of this project (Turc et al, 2021; doi: 10.3389/fpls.2021.679852), recording 10 measurements per leaf. Although this second experiment was conducted in the exact same conditions as a previous one described in Turc et al (2021), chlorophyll content decreased earlier when using biochemical assay. Leaves of our poplar were very big, therefore biochemical estimation of chlorophyll content on homogenized half leaf might be more accurate than reflectance-based method (even if we did 10 records per leaf).

Best regards,

Mr Benjamin Turc
